# Ambient aerosols increase stomatal transpiration and conductance of hydroponic sunflowers by extending the hydraulic system to the leaf surface

**DOI:** 10.3389/fpls.2023.1275358

**Published:** 2023-11-30

**Authors:** Juergen Burkhardt, Daniel Zinsmeister, Anita Roth-Nebelsick, Hubert Hüging, Shyam Pariyar

**Affiliations:** ^1^ Institute of Crop Science and Resource Conservation, Plant Nutrition Group, University of Bonn, Bonn, Germany; ^2^ Department Palaeontology, State Museum of Natural History Stuttgart, Stuttgart, Germany; ^3^ Institute of Crop Science and Resource Conservation, Crop Science Group, University of Bonn, Bonn, Germany

**Keywords:** anisohydric, atmospheric drought, VPD, hydraulic activation of stomata, leaf hydraulics, proline, TPU limitation, wick

## Abstract

**Introduction:**

Many atmospheric aerosols are hygroscopic and play an important role in cloud formation. Similarly, aerosols become sites of micro-condensation when they deposit to the upper and lower surfaces of leaves. Deposited salts, in particular can trigger condensation at humidities considerably below atmospheric saturation, according to their hygroscopicity and the relative humidity within the leaf boundary layer. Salt induced water potential gradients and the resulting dynamics of concentrated salt solutions can be expected to affect plant water relations.

**Methods:**

Hydroponic sunflowers were grown in filtered (FA) and unfiltered, ambient air (AA). Sap flow was measured for 18 days and several indicators of incipient drought stress were studied.

**Results:**

At 2% difference in mean vapor pressure deficit (D), AA sunflowers had 49% higher mean transpiration rates, lower osmotic potential, higher proline concentrations, and different tracer transport patterns in the leaf compared to FA sunflowers. Aerosols increased plant conductance particularly at low D.

**Discussion:**

The proposed mechanism is that thin aqueous films of salt solutions from deliquescent deposited aerosols enter into stomata and cause an extension of the hydraulic system. This hydraulic connection leads – parallel to stomatal water vapor transpiration – to wick-like stomatal loss of liquid water and to a higher impact of D on plant water loss. Due to ample water supply by hydroponic cultivation, AA plants thrived as well as FA plants, but under more challenging conditions, aerosol deposits may make plants more susceptible to drought stress.

## 1 Introduction

Atmospheric vapor pressure deficit (D) is the driver of plant transpiration (E) and is determined by air temperature (T) and relative humidity (RH). Given the exponential T dependence of the saturation vapor curve, D has been rising exponentially worldwide due to climate change and has become a major factor in recent drought-induced plant mortality (Novick et al., 2016; Grossiord et al., 2020). Apart from driving E, the increasing D also acts as a stimulus to close stomata, as seen from the direct closing response of stomatal guard cells to decreasing air humidity (Lange et al., 1971; Bauer et al., 2013). Responses of leaf conductance to increasing D generally follow a hyperbolic or exponential decrease, while the magnitude of the decrease has been used to describe the stomatal sensitivity (Leuning, 1995; Oren et al., 1999; Medlyn et al., 2011). The mechanistic basis of this reaction has been subject to a number of theories regarding the stimulus factor (RH or D), the existence and site of a ‘humidity sensor’, the reaction type (feedback, feedforward), and the involvement of hormonal and genetic factors (Farquhar, 1978; Ball et al., 1987; Grantz, 1990; Monteith, 1995; Xie et al., 2006; Bauer et al., 2013; Cardoso et al., 2020). The actual site of evaporation, i.e., the end of the plant´s hydraulic system, might also play an important role. The path of the water molecules from leaf xylem to the atmosphere remains difficult to measure experimentally and has been subject to contrasting model interpretations (Farquhar and Raschke, 1978; Tyree and Yianoulis, 1980; Boyer, 1985; Rockwell et al., 2014; Scoffoni et al., 2017), with the role of bundle sheath extensions and stomatal subsidiary cells, and the relative importance of liquid and vapor transport all awaiting clarification. The humidity within the substomatal cavity (Cernusak et al., 2018; Wong et al., 2022) and the primary site of evaporation are not clear and may also vary among species and environments (Buckley, 2015; Scoffoni, 2015).

So far, gas exchange theories and interpretations have usually considered the leaf surface as a passive diffusion barrier and ignored the possible effects of deposited hygroscopic aerosols. Atmospheric aerosols are ubiquitous; most of them are water soluble salts or otherwise hygroscopic and thus play a decisive role in cloud formation (Petters and Kreidenweis, 2007). After their deposition on leaf surfaces, aerosols may similarly interact with transpired water from the plant, a fact that has received little attention. Salts have been used to measure relative humidity (RH) in radiosondes and to achieve constant RH in closed environments, due to their rapid and reproducible equilibration with surrounding water vapor by condensation and evaporation (Wylie, 1955; Winston and Bates, 1960). Once local RH exceeds the salt specific deliquescence humidity (DRH), e.g. 75% RH for NaCl, the equilibrium state of a salt is a solution droplet rather than a crystal (Pilinis et al., 1989). The recognition of deposited aerosols on leaf surfaces is complicated, because transpiring stomata and the humid boundary layer foster deliquescence. The micro-condensation to leaf surface particles can be visualized using environmental scanning electron microscopy (ESEM; Eiden et al., 1994; Burkhardt and Hunsche, 2013). In ambient outdoor environment, the resulting salt solution films can be detected by the high correlation of electrical leaf surface conductance with atmospheric RH, e.g., on spruce needles and potato leaves, even on hot, sunny days (Burkhardt and Eiden, 1994; Burkhardt and Hunsche, 2013).

While the formation of thin films of concentrated salt solution appears to be a feature of the natural environment (Burkhardt and Grantz, 2017), most meteorological and ecophysiological approaches do not consider micro-condensation to deposited aerosols and assume 100% local RH as a requirement for the formation of dew or condensation (e.g., Agam and Berliner, 2006). The resulting bias is relatively small in meteorological contexts focusing on water quantities. But for plant water relations, the microscopic interaction of concentrated salt solutions with water vapor and leaf surfaces may be highly relevant. Concentrated salt solutions can react dynamically to humidity fluctuations by repeated deliquescence/efflorescence cycles causing salt creep (Qazi et al., 2019), and ESEM studies confirmed that such creeping salt solutions can enter into stomatal structures (Burkhardt and Hunsche, 2013). Once having entered into the substomatal cavity via the cuticular surface of the guard cell walls, the solution films may connect to the apoplastic water that is usually considered to form the end of the plant hydraulic system (i.e., the site of evaporation within the leaf). Such a connection would establish a thin and probably persistent wick with its end on the leaf surface, which is expected to be able to shift the site of evaporation to the leaf surface, and to increase transpiration (Burkhardt, 2010).

The hypothetical establishment of such an aqueous connection (‘hydraulic activation of stomata’ – HAS; Burkhardt, 2010) is hindered by the hydrophobic cuticle covering the leaf surface and partially the stomatal walls (Nonami et al., 1991), and this resistance has to be overcome for each single pore. There have been several direct and indirect proofs of water, ion, and nanoparticle transport into the stomata (Eichert et al., 2008; Arsic et al., 2020; El-Shetehy et al., 2021). HAS establishment is particularly feasible for concentrated solutions of chaotropic salts from the Hofmeister series, which have lower surface tension and can overcome the usual hydrophobicity of leaf surfaces and internal cuticles (Pegram and Record, 2007; Roth-Nebelsick, 2007; Tobias and Hemminger, 2008; Kunz, 2010; Burkhardt et al., 2012). The efficacy of the Hofmeister series in this regard requires high ionic strength of the respective solution (> 0.1 M). Such conditions are common on the leaf surface, where deliquescent salts are close to saturation. Chaotropic salts or acids seem to work best for chemical desiccation, used in plant breeding and practical agriculture (Murphy, 1968; Blum et al., 1983; Nicolas and Turner, 1993; Burkhardt, 2010; Burkhardt et al., 2012), and deliquescent chaotropic salt solutions like potassium iodide (KI) or potassium thiocyanate (KSCN) were observed to readily enter into stomatal pore structures (Burkhardt and Hunsche, 2013; ESEM movies in supplementary material). For kosmotropic salts like NaCl, this process is less effective but can be supported by surfactants, which has been considered causal for the decline of sea spray tolerant coastal forests in the vicinity of landfills in Australia and Italy (Grieve and Pitman, 1978; Bussotti et al., 1995). The creeping process is supported by repeated deliquescence and efflorescence of salts and thus driven by local RH fluctuations (Qazi et al., 2019; Ivanova and Esenbaev, 2021) that are continuously occurring due to temperature changes and sunflecks. The HAS process is of a physical nature, although potassium salts might interact with guard cell physiology (Kollist et al., 2014; Wang et al., 2017).

There has been some support for such a mechanism, when salt solutions sprayed onto beech seedlings increased sap flow rates. Pine seedlings, however, kept transpiration stable and showed a tendency to reduce photosynthesis (Burkhardt and Pariyar, 2016). In an aerosol exclusion experiment with Faba bean, aerosol reduction increased stomatal aperture at equivalent flux, reduced nocturnal water vapor flux and minimum leaf conductance, and increased heterogeneity of stomatal aperture across the leaf surface (patchiness) compared to plants in unfiltered air (Grantz et al., 2018; Grantz et al., 2020). The response of plants to hygroscopic substances on leaves may thus depend on the respective isohydric or anisohydric strategy of the plant, while small effects and low transpiration rates may cause difficulties in interpretation (Burkhardt et al., 2001; Pariyar et al., 2013; Chi et al., 2022).

Here, we hypothesized that ambient aerosols influence the transpiration of hydroponic sunflowers, and addressed this with an aerosol exclusion experiment. Sunflower (*Helianthus annuus*), an important oil crop with high transpiration rates is considered an anisohydric plant species with little stomatal response to D and high transpiration rates due to an efficient hydraulic system (Turner et al., 1985; Tardieu and Simonneau, 1998). Still, preliminary aerosol exclusion studies with sunflowers indicated an influence of the aerosol environment on the leaf conductance response to increasing D within a ventilated cuvette (Pariyar et al., 2013). In the current study, transpiration measurements were conducted using the sap flow method, i.e., with undisturbed leaf boundary layer. Sunflowers were supplied with optimum water and nutrient conditions, and exposed to different aerosol environments in greenhouses, one group receiving unfiltered, ambient air (AA), the other one receiving filtered air with less than 2% of original aerosols remaining (FA). 18 days of parallel AA and FA transpiration measurements were complemented by the determination of several water status parameters and potential indicators of incipient water deficit, including gas exchange, osmotic adjustment, and leaf hydraulics. Hydroponic cultivation avoided water supply issues and enabled the concentration on plant/atmosphere interaction.

## 2 Materials and methods

### 2.1 Plant material and growth environment

Sunflower (*Helianthus annuus* var. Olmedo) seeds were first sown on sand for germination then transplanted to a hydroponic system with four seedlings per 10 L pot at the 2-leaf stage (10 days after planting, DAP). Seedlings were randomly assigned to one of two adjacent greenhouses, located within the urban area, near a multi-lane highway in Bonn, Germany. One greenhouse was supplied with ambient air (AA), the other one with filtered air (FA), from which nearly all particles were excluded. For both greenhouses, complete air changes took place at a rate of two times min^-1^. Filtration of FA to HEPA standards was achieved with a cloth bag followed by high efficiency filter pad (H 13; ACS; Essen, Germany; Burkhardt and Pariyar, 2016). During the sunflower growth period, typical ambient aerosol concentrations below 10 µm in aerodynamic diameter (PM10) at the closest monitoring station (Bonn-Auerberg; DENW062; LANUV Essen, 2017) were 22 ± 9 µg m^-3^ (mean ± standard deviation). Within the greenhouses, total aerosol number concentrations (> 10 nm), measured with a cloud chamber condensation nuclei counter (TSI 3783; TSI, Shoreview, MN, USA) was 6 – 7 x 10^9^ particles m^-3^ in AA and reduced to 5-10 x 10^6^ m^-3^ in FA i.e. by > 99% as previously reported (Grantz et al., 2018). The FA aerosol mass concentration was 19% of AA levels for NH_4_^+^, 33% (Na^+^), 13% (NO_3_^-^), 17% (Cl^-^), and 6% (SO_4_^2-^) (Burkhardt et al., 2018). FA and AA did not differ for HCl, SO_2_, and NH_3_, while FA was 19% higher than AA for HNO_3_ (Burkhardt et al., 2018). Typical hourly and annual trends of ozone (O_3_) concentrations in the greenhouses were similar in the two greenhouses, with O_3_ below 35 ppb and a few ppb lower in FA than in AA due to aerosol filtration (Grantz et al., 2018).

**Figure 1:**
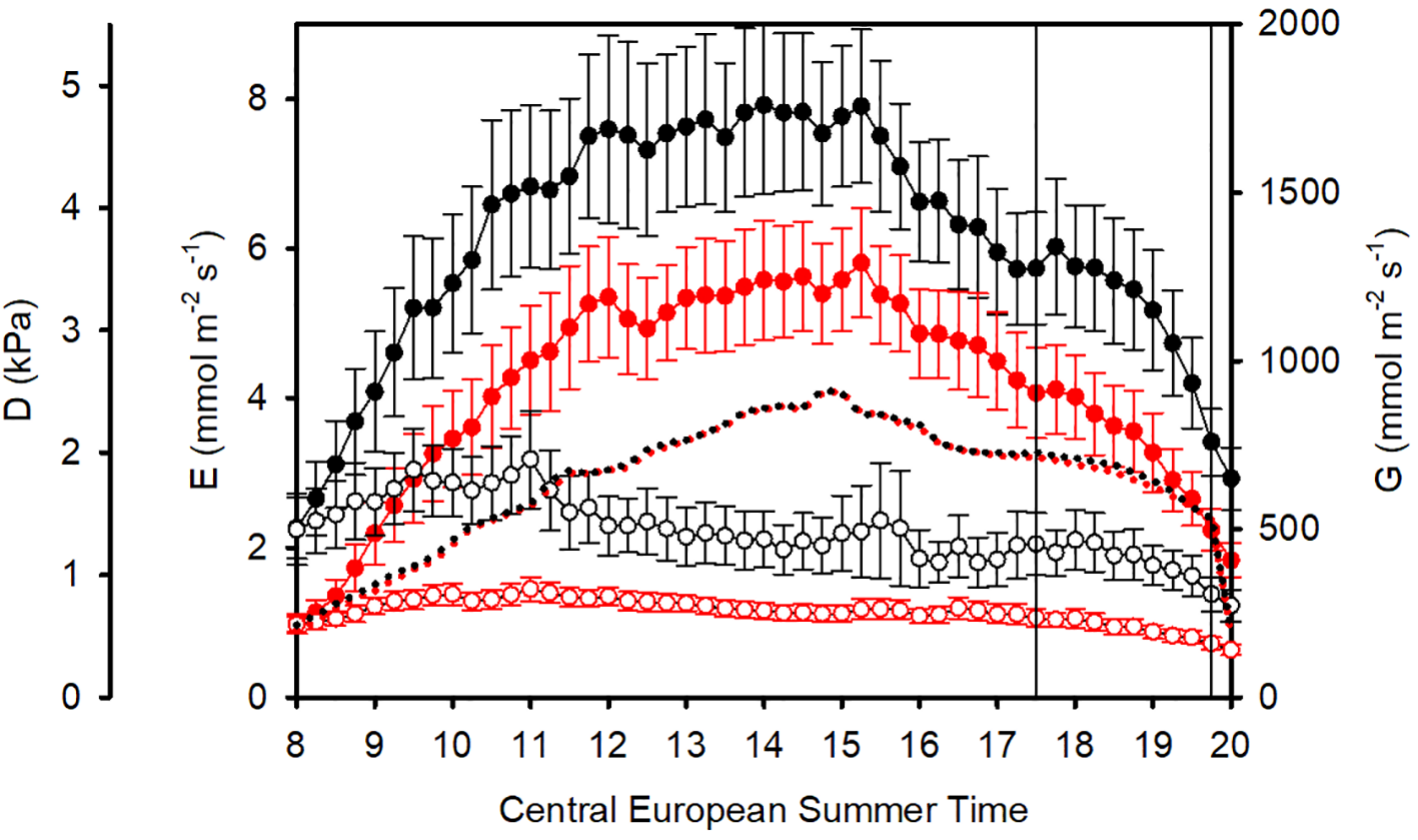
Mean diurnal courses from 8 h to 20 h (CEST): Vapor pressure deficit D (dotted), sunflower transpiration rates E (filled symbols with standard errors), and plant conductances G (empty symbols straight lines with standard errors). Black: unfiltered, ambient air (AA). Red: filtered air (FA). Data are averaged from 18 days of measurements. Sap flow data between 17:30 h and 19:30 h (vertical lines) were not considered in further data analysis due to differences in D, caused by different shadowing of the greenhouses.

Greenhouses were oriented parallel to each other, so the light environment was the same, except for the shadow of a tree, which briefly reduced temperatures and D on sunny afternoons (17:30 h– 19:30 h) in FA (vertical lines, **Figure 1**). These data were excluded from analysis. Plants were exposed to natural daylength and sunlight (up to 1500 µmol m^-2^ s^-1^ at plant level; 70% of ambient near midday). Temperature (T) and relative humidity (RH) were recorded every 15 minutes and used to calculate vapor pressure deficit (D). Highest D levels reached 5.9 kPa (c. 41°C and 26% RH) on a 15-minute basis and 3.2 kPa on a daily basis (8:00 h to 20:00 h). The hydroponic nutrient solution contained all essential macro- and micronutrients with continuous aeration. The solution was changed one to three times week^-1^ depending on the growth stage. Plants grew to c. 2 m height and flowered at c. 50 DAP, with 11 to 12 leaf pairs.

Using paired measurements on eight AA and FA plants, sap flow, leaf water potential, osmotic potential, proline, foliar chlorophyll content, leaf mass at harvest, leaf mass per area, and foliar carbon isotopes were measured. In addition, gas exchange measurements were performed and the fluorescein tracer distribution within cut leaves was microscopically analyzed.

### 2.2 Sap flow and leaf mass per area

Sap flow rates of the sunflowers were measured with a heat balance system (32A, Dynamax Inc., Houston, USA), with a constant heat source surrounding the shoot. Transpiration removes heat proportionally to the flow rate. Sap flow sensors were installed when plants had grown to 50 – 70 cm (39 DAP) and were continuously monitored for 18 days (39-56 DAP). Plants were harvested on day 57 DAP. Gauges were covered by radiation shields to protect against radiation and accidental wetting during irrigation. The system was powered down at night (23:00 h until 4:00 h) to prevent the stem from overheating. Data were checked daily and adjustments made to account for stem growth. The sap flow was then computed, using the suggested quality control procedures from the manufacturer. Data analysis focused on sap flow data measured between 8:00 h and 20:00 h Central European Summer Time (CEST). During this time period, transpiration was usually tightly coupled to D. 

After finishing the sap flow measurements, all leaves were collected and the total leaf area of the plants was quantified using a leaf area measuring system, based on a commercial scanner with specifically adapted software (OMA, HGoTech, Bonn, Germany). The dry mass of leaves was determined after five days at 60°C, and related to leaf area to determine leaf mass per area (LMA). For the calculation of leaf area related sap flow data, a daily increase of 9.2% in leaf area was used based on leaf growth rate data for hydroponic sunflower presented by Rivelli et al., (2010), calculating back from the harvested leaf area of each plant. For each 15 min time interval, replicate plants (N=8) were averaged and related to the calculated D of the corresponding greenhouse. From transpiration rates (mmol m^-2^ s^-1^) and D values, 15-minute values of plant conductances were determined as G = P * E/D, where G is plant conductance (mol m^-2^ s^-1^) and P is atmospheric pressure (Pa).

### 2.3 Gas exchange

Gas exchange was measured (33-34 DAP; N = 8), using a steady state gas exchange system (6400XT; LI-COR Biosciences Inc., Lincoln, NE, USA). Light curves and A/Ci curves were measured at 30°C leaf temperature inside the greenhouses, where lower temperatures could not be stabilized. Light curves were derived from measurements at 400 ppm CO_2_, leaf temperature (T_leaf_) of 30°C and D of c. 1.7 kPa and flow rate of 500 µmol s^-1^, while photosynthetically active radiation (PAR) inside the chamber tracked outside PAR over a period of about five hours. Data were then fitted by a three parameter, exponential rise to maximum equation and averaged between replicate plants. From the resulting equations, maximum net assimilation rate Amax, light compensation point and dark respiration rate were derived. Highest light levels in the greenhouses were 1400 μmol m^−2^ s^−1^, and at this point A reached 90% of the maximum value extrapolated to fully saturating 2000 µmol m^-2^ s^-1^. A/Ci curves were measured with constant light at 1400 μmol m^−2^ s^−1^, 30°C leaf temperature, and flow of 300 µmol s^−1^. The duration of each step of CO_2_ concentration was between 120 and 180 s depending on stability. CO_2_ concentrations started from 400 ppm, then progressed to 250, 150, 100, 50, 400, 400, 600, 800, 1000, 1200, 1600, 2000. After correcting primary data for leaks using the routine suggested by the manufacturer, data from A/Ci curves were used to calculate maximum carboxylation rate of Rubisco (V_cmax_), maximum rate of electron transport for the given light intensity (J_max_) and carboxylation rates limited by triose phosphate utilization (TPU), using the *plantecophys* package in R Studio (R; v. 4.0.3; Duursma, 2015), with fixed daytime respiration (R_d_) of 1.5 μmol m^−2^ s^−1^ (Sun et al., 2023).

### 2.4 Leaf water potential (Ψ), osmotic potential, proline and chlorophyll content

Leaf water potential (Ψ) was measured with a Scholander Pressure Chamber (Soil Moisture Equipment Corp., Santa Barbara, CA, USA). Ψ was measured at DAP 57 at 4 pm (D: 4.8 kPa) because the highest D in the greenhouses was normally reached at this time of the day. For osmotic potential, proline and chlorophyll content analysis, 5^th^ leaf pair samples were taken. Leaf samples were stored at -20°C until the analysis was carried out. About 4 g of leaf fresh mass (FM) from each sample was dipped in liquid nitrogen (-200°C) and was squeezed. The extract (200 μl) was centrifuged at 10,000 rpm for 10 min at 4°C. Osmotic potential (Ψπ) of 15 μl supernatant was analyzed twice (Osmomat 030-D, Gonotec GmbH, Berlin, Germany). Osmotic potential mean values [osmol kg^-1^] were multiplied by -2.437 (correction coefficient valid for 20°C) to get Ψπ in MPa (Taiz and Zeiger, 2010). The leaf samples were freeze dried (Gamma 1-16 LSC, Martin Christ GmbH, Osterode, Germany) and were ground for determinations of proline and chlorophyll contents. The proline extraction was done according to the procedure of Pariyar and Noga, (2018), using sulfosalicylic acid, glacial acetic acid (100%) and ninhydrin acid reagent. Absorbance of the extracted solution (red colored organic phase) was measured with a spectrophotometer (Lambda 35 UV/VIS Spectrophotometer, PerkinElmer, MA, USA) at a wavelength of 520 nm. Chlorophyll extraction was done using methanol as solvent and absorbance of the extract measured at 650 nm and 665 nm wavelengths to calculate the total chlorophyll content in the leaf samples (Pariyar and Noga, 2018).

### 2.5 Foliar carbon isotope concentrations δ^13^C

Samples for analysis of carbon isotope composition were analyzed for leaves from different stages (old leaves: 4th leaf pair; young leaves: 10^th^ leaf pair). δ^13^C was measured with an isotope ratio mass spectrometer (IRMS, Sercon Ltd, Cheshire, UK) as described earlier (Burkhardt and Pariyar, 2016). The carbon isotope composition (δ^13^C) was calculated by comparison to a standard. Air samples of the AA and FA greenhouse air were taken and δ^13^C measured at Max-Planck-Institute for Biogeochemistry, Jena, Germany. The results showed that filtration had no effect on carbon isotopes in incoming air.

### 2.6 Leaf hydraulics/fluorescence

A leaf from the 7^th^ leaf pair from each plant (N = 5 for each treatment) was cut from the plant in the morning (approx. 10 AM) and the petioles were immediately (< 3 sec) transferred into pure water. After collecting all leaf samples, the petiole cut ends were trimmed under water to avoid embolism and immediately placed into a 2.8 mM sodium fluorescein solution. All leaf samples were left to transpire for 20 minutes in the same growth environment. The petiole of each leaf was then sealed with paraffin and aluminium foil and placed in Petri dishes which were then sealed. The samples were kept in darkness until fluorescence images were recorded with a Multispectral Fluorescence Imaging System (Nuance® TM, PerkinElmer, MA, USA), integrated with a Stereomicroscope (Carl Zeiss MicroImaging GmbH, Jena, Germany). Fluorescence images were recorded in the dark at 22°C by using the full CCD frame (1392 × 1040 pixels), with a 0.8x Zeiss Neo Lumar objective, 11x magnification with an object field of 124 mm^2^ with Lumar filter 09FITC at 530 nm (green, fluorescein emission). Brightness and contrast were held constant for all images.

### 2.7 Statistics

Individual plants were taken as replicates (N = 8). For the sap flow measurements, mean 15-minute transpiration rates were calculated from the number of respective repetitions, and the data was pooled for AA or FA transpiration rates of both sunflower generations. Statistical analysis was performed and graphics were prepared using Sigmaplot v14 (Systat Software GmbH, Germany). One-way analysis of variance (repeated measures one-way ANOVA) was performed for normally distributed data. The significance was estimated between groups by pairwise comparisons using the Student-Newman-Keuls (SNK) and Holm-Sidak Test. When these tests failed due to the different sampling numbers, Dunn´s method was used. When the data were not normally distributed, the statistical analysis was performed with a non-parametric method using the Kruskal-Wallis-Test to see the interactive group effect, and the two-sided Mann-Whitney-U-test for differences between two groups. The daily course of D and E was compared by paired t-tests for simultaneous AA and FA measurements. Differences were considered significant if p < 0.05. Additionally, linear regression was performed to detect the correlation between atmospheric vapor pressure deficit (D) and plant transpiration (E), and hyperbolic decay regression for the D dependence on plant conductance (G), in each treatment environment (i.e., AA or FA).

## 3 Results

### 3.1 Sap flow

On a daily basis (from 8:00 h to 20:00 h, excluding 17:30 h to 19:30 h), the overall mean vapor pressure deficit in AA was D = 1.712 kPa and in FA D = 1.683 kPa, which was 1.7% higher in AA than FA (**Table 1**). The daily D course of 15-minute means for all 38 days is shown in **Figure 1** (dotted curves). Both transpiration fluxes and conductances were higher in AA (black) than in FA (red) throughout the day.

**Table 1:** Environmental and physiological parameters, compared between the two greenhouses (AA and FA), with exact P-values giving the level of significance, and * (< 0.05), ** (< 0.01), *** (< 0.001), ns, not significant giving the category.

Parameter	AA	FA	Statistics
Mean vapor pressure deficit D (kPa) (15-minute values)	1.712	1.683	r^2 =^ 0.997 ***(P < 0.001)
Mean transpiration rate E (mmol m^-2^ s^-1^) (15-minute values)	6.041	4.062	r^2 =^ 0.760 ***(P < 0.001)
Mean conductance G (mmol m^-2^ s^-1^) (15-minute values)	500	257	r^2 =^ 0.705 ***(P < 0.001)
E dependence on D (mmol m^-2^ s^-1^) (15-minute values)	E_AA_ = 3.58 + 1.44 * D_AA_	E_FA_ = 0.82 + 1.93 * D_FA_	AA: r^2 =^ 0.18 FA: r^2 =^ 0.59
G dependence on D (mmol m^-2^ s^-1^) (15-minute values)	G_AA_ = -1387 + 2.4E4/(11.3 + D_AA_) Linear: G_AA_ = 733 - 136 * D_AA_	Linear: G_FA_ = 284 - 15.7 * D_FA_	AA: r^2 =^ 0.16 Linear AA: r^2 =^ 0.16 Linear FA: r^2 =^ 0.03
δ^13^C young leaves old leaves	-30.58 ± 0.11 -30.86 ± 0.14	-30.10 ± 0.07 -31.02 ± 0.16	** (P = 0.001) ns (P = 0.461)
Leaf area (m^2^)	0.151 ± 0.009	0.169 ± 0.009	ns (P = 0.200)
Leaf mass per area LMA (g m^-2^)	34.9 ± 0.5	31.8 ± 0.7	**(P = 0.004)
Water potential (MPa), 4 pm (D=4.8 kPa)	-0.556 ± 0.052	-0.539 ± 0.063	ns (P = 0.432)
Osmotic potential (MPa)	-1.01 ± 0.02	-0.96 ± 0.02	* (P = 0.024)
Proline (µmol g^-1^)	187 ± 11	154 ± 6	*(P = 0.013)
Chlorophyll (mg g^-1^)	15.41 ± 0.24	14.95 ± 0.42	ns (P = 0.382)
Max net assimilation rate (µmol m^-2^ s^-1^ CO_2_) Light compensation point (µmol m^-2^ s^-1^ PAR) Dark respiration rate (µmol m^-2^ s^-1^ CO_2_)	61.21 ± 3.20 57.63 ± 6.07 -4.15 ± 0.91	69.25 ± 7.77 53.83± 3.86 -3.89 ± 0.55	ns (P = 0.327) ns (P = 0.587) ns (P = 0.801)
Vcmax (µmol m^-2^ s^-1^) Jmax (µmol m^-2^ s^-1^) TPU (µmol m^-2^ s^-1^)	146 ± 7 541 ± 97 18.3 ± 0.6	156 ± 6 806 ± 152 19.9 ± 1.3	ns (P = 0.275) ns (P = 0.174) ns (P = 0.055)

**Figure 2:**
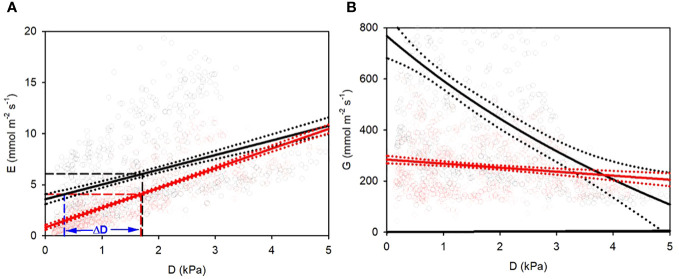
**(A)** Transpiration E and **(B)** conductance G response of hydroponic sunflowers to vapor pressure deficit D. Black: AA plants, red: FA plants. **(A)** linear regressions (straight lines) with 95% confidential range (dotted lines) for E-D relationships. Dashed vertical and horizontal lines indicate overall mean values of D and E. AA plants need less D to reach equal transpiration with FA plants, as shown by ΔD for the overall mean of FA. **(B)** regressions for hyperbolic (AA) and linear (FA) decay of G-D relationships. Dotted lines: 95% confidence band of regression curves.

**Figure 2A** shows the correlations between D and E, based on the 679 data point pairs (AA: black; FA: red, respectively) from all 15-minute intervals. Each data point represents the transpiration mean of all recorded plant individuals for the respective time interval, i.e., the mean of 8 biological repetitions.

The overall AA and FA regressions yielded higher threshold and lower slope for AA compared to FA plants (**Figure 2A**; **Table 1**). The transpiration of AA sunflowers reached the average transpiration rate of FA sunflowers 4.07 mmol m^-2^ s^-1^ at 0.34 kPa, i.e., 1.34 kPa lower than FA, indicated by the blue vertical line and ΔD (**Figure 2A**). FA sunflowers reached the average transpiration rate of AA sunflowers only at 3.55 kPa, i.e., 1.84 kPa higher than AA (not shown). FA transpiration rates were more strongly correlated with D (r^2 ^= 0.59 for 15-minute-means) compared to AA plants (r^2 ^= 0.16; **Table 1**), which suggests additional contributing factors for AA. The mean G value for AA plants was about twice as high as for FA plants (500 and 257 mmol m^-2^ s^-1^, respectively) and G remained higher for AA than FA for D < 3.1 kPa (**Figure 2B**; **Table 1**). G was almost completely independent of D for FA (r^2 ^= 0.03) but strongly reduced by increasing D in AA. The dependence of G on D was nearly linear rather than hyperbolic (**Figure 2B**; **Table 1**).

### 3.2 Foliar concentrations, water potential and photosynthesis

None of the plants showed visual signs of drought, but some agronomic and physiological measurements revealed effects of incipient water deficit of AA plants compared to FA. In many plant traits and measured parameters, AA and FA plants did not differ (**Table 1**). The overall evaluation of the experiment showed no differences in leaf area. Neither the chlorophyll content nor leaf water potentials were different. Interestingly, the LMA of AA leaves was significantly higher (p = 0.004) compared to FA leaves.

Carbon isotope ratio (δ^13^C) values of young FA leaves were less negative than AA, indicating stomata were less open in FA plants under similar environmental conditions. Older leaves did not show differences between AA and FA (**Table 1**).

Leaf water potential (Ψ) did not differ between AA and FA plants. The osmotic potential, however, was more negative for AA than for FA leaves, and proline concentration was higher for AA than for FA leaves (**Table 1**).

**Figure 3:**
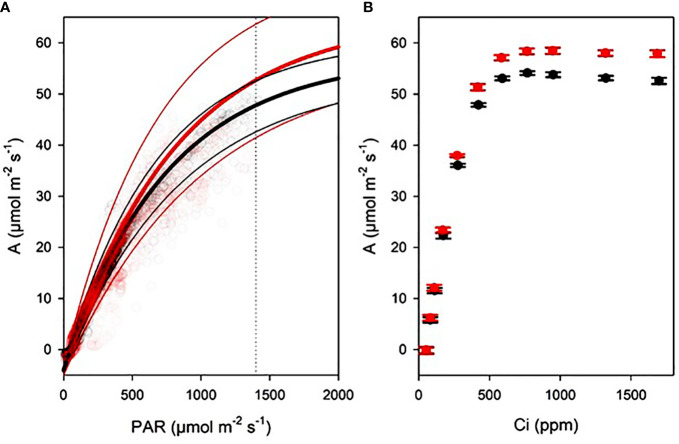
Gas exchange curves of AA (black) and FA (red) sunflowers N = 8. **(A)** Light curves (thick lines) with standard errors (thin lines), with exponential maximum curves derived from continuous gas exchange with outside light tracking. Dashed line indicates light level used for CO_2_ concentration curves. **(B)** CO_2_ concentration curves with data points and standard errors, measured with an A/Ci routine.

The light curves resulted in A = -4.153 + 69.25*(1-exp(-0.00123*PAR)) for FA and A = -3.894 + 61.21*(1-exp(-0.00133*PAR)) for AA. Maximum photosynthesis, light compensation point, and dark respiration rate were not significantly different between AA and FA plants (**Figure 3A**; **Table 1**). Similarly the A/Ci curves did not reveal significant differences, although TPU limitation difference between AA and FA plants was close to significance (P = 0.055; **Figure 3B**; **Table 1**).

**Figure 4:**
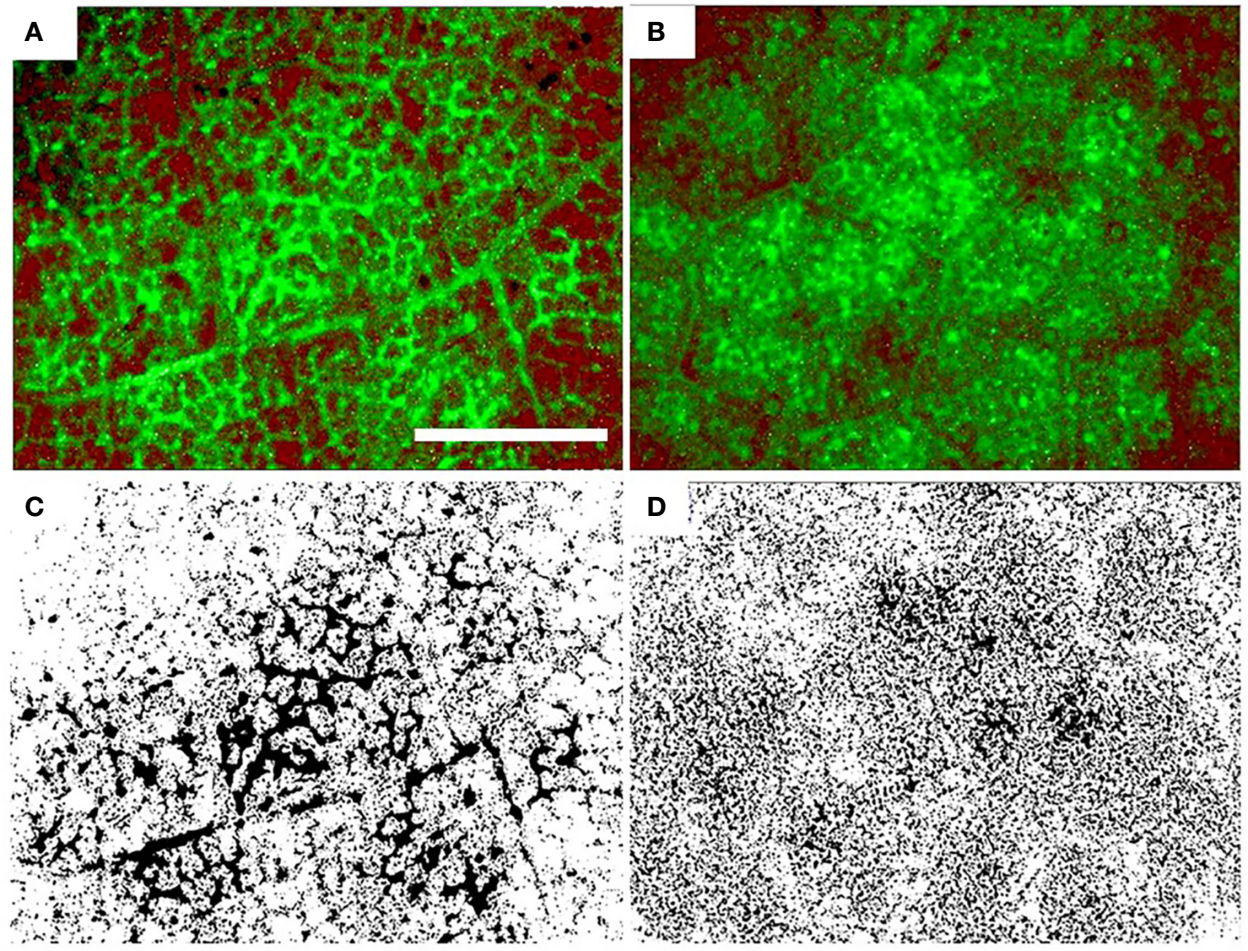
Distribution of fluorescein in sunflower leaves grown in ambient air **(A, C)** and filtered air **(B, D)**. The bottom row is a black and white version of the top row. Fluorescein appears as a green color in the top row and as black in the bottom row. Scale bar: 3 mm.

AA and FA leaves showed different fluorescence patterns after introduction of sodium fluorescein in the transpiration stream. In AA leaves, the tracer was localized in small veins with little green color visible outside of the veins (**Figures 4A, C**). In FA leaves, uniformly distributed green color was seen in ‘cloud-like’ areas (**Figures 4B, D**) in the areole area outside of the ultimate veins. It was not possible to distinguish if the dye was in the apoplast or in the symplast.

## 4 Discussion

Higher transpiration flux and conductance of AA compared to FA plants confirmed the results of a previous experiment with hydroponic sunflowers in filtered and unfiltered ambient air (Pariyar et al., 2013). The differences were in the same range: 49% higher mean AA transpiration compared to FA means 33% lower FA transpiration compared to AA (the previous study had considered FA as the experimental treatment and AA as the control). The largest conductance (G) differences between AA and FA were observed at low D and the difference decreased with increasing D, as previously described (Pariyar et al., 2013; Grantz et al., 2018). Also, a decrease of the osmotic potential with aerosol was confirmed, though values were higher than previously observed, and was supported by elevated proline concentrations in AA plants observed in the present study.

Not unexpectedly, the absolute values of transpiration and conductance were lower in this study compared to the previous results (Pariyar et al., 2013), due to the differences in measurement conditions: here, transpiration was measured using the sap flow method and thus plants with intact, undisturbed leaf boundary layers, while the previous study had used a highly ventilated cuvette system (Pariyar et al., 2013). Different from the previous study, which reported lower water potential of AA leaves, the AA and FA leaf water potentials were now statistically indistinguishable. However, in spite of hydroponic water supply, AA leaves showed stronger signs of incipient local or long-term drought than leaves: including a higher proline content, more negative osmotic potential, higher LMA, and different water distribution in the apoplast. Higher proline concentration and lower osmotic potential are generally observed long-term acclimation reactions, including for sunflowers (Manivannan et al., 2007; Fulda et al., 2011; Cardoso et al., 2018). Similarly, LMA increased under drought for *Amaranthus* spp. (Liu and Stützel, 2004), though for sunflower the higher LMA of the AA leaves might be confounded by seasonal and light-induced changes (Ratjen and Kage, 2013; Miner and Bauerle, 2019).

With AA and FA plants experiencing equal, optimum water supply and almost equal atmospheric demand D during the measurements, it is reasonable to assume that differences in transpiration rates are a consequence of an aerosol influence on plant conductance. Deposited aerosols may change the respective efficiencies of water transport along the known hydraulic (liquid water) or gaseous (water vapor) pathways, or cause changes in the structure of these pathways. Plant conductance to water transport is composed of the liquid water pathway, characterized by the hydraulic conductance of roots, shoot, and leaves, and the water vapor pathway, characterized by the conductance of stomata, cuticle, and leaf boundary layer. The contribution of cuticular conductance is usually low (about 0.5 mmol m^-2^ s^-1^ on average, Duursma et al., 2019) and can be neglected under daytime conditions. Atmospheric aerosols, which are a mixture of many different chemical substances, could change the conditions not only by the physicochemical properties such as hygroscopicity and deliquescence, but also by chemical interaction, e.g., by making the cuticle more permeable to water vapor. Because i) the additional conductance diminishes at higher D and ii) earlier studies had reported that salt spray to beech leaves had increased the transpiration similarly (Burkhardt and Pariyar, 2016), it seems unlikely that such an effect on the cuticle was the major reason here. The conductance of the leaf boundary layer depends on the ventilation and position of plants and leaves in the greenhouse. Ventilation was similar between the greenhouses and plants were similarly positioned. Thus, differential influence of the leaf boundary layer seems to be excluded as a major reason for the average 49% higher transpiration rates of AA compared to FA plants.

In general and with regard to the hydraulic part of the water transport system, the phenological stage and plant size did not differ between AA and FA plants. Plants in both groups thrived equally well, and there were no obvious differences in the hydraulic architecture and properties of roots and shoots. But the additional demand on the water transport system in the AA plants, caused by the higher transpiration rate, may have induced additional anatomical effects, for instance, altered stomatal density, which can be affected by air humidity and drought under systemic signaling from older leaves (Santrucek et al., 2014; Monclus et al., 2006). These were not measured here.

The overall leaf water potential did not differ between AA and FA, but it is known that water potential can be heterogeneous in large leaves (Li et al., 2013). Continuously higher transpiration rates and possibly local supply deficiencies within smaller veins could have triggered proline synthesis for osmotic protection, without measurable differences in overall leaf water potential. The difference in dye distribution patterns of infiltrated AA vs. FA leaves is a visible impact of aerosols on plant hydraulics and showed remarkable similarity with observations on droughted vs. well-watered sunflower leaves (Trifilo et al., 2003). Differences between AA and FA dye distribution patterns may reflect shifts in water transport pathways. For instance, it might be possible that the sites of evaporation are different for AA plants and FA plants, and/or that partitioning between apoplastic and symplastic transport of liquid water is different (Cernusak et al., 2018; Grossiord et al., 2020; Wong et al., 2022). This is, however, conjectural at present and might merit further studies.

Direct, relevant interaction of aerosols with plant water can be expected in the stomatal region. Here, two possible factors can be influenced: stomatal aperture, which according to common concepts is the responsible factor of stomatal conductance (Parlange and Waggoner, 1970), and the transition from the liquid phase to water vapor, taking place at the end of the hydraulic transport system. Both factors could theoretically be influenced by aerosols.

Wider stomatal apertures of AA leaves due to the presence of aerosols could be judged by the comparison of isotopic δ^13^C determination of leaves. AA and FA plants were grown under the same environmental conditions, except for aerosols, which should enable the detection of long-term differences in stomatal opening (Farquhar and Richards, 1984). According to the more negative δ^13^C of young AA leaves, their stomata were more open, allowing higher discrimination of ^13^CO_2_ compared to FA leaves of the same age. Although the plants were fully nourished from the nutrient solution, locally supplied nutrients from aerosols to the leaf surface might have fostered an increase of stomatal conductance or affected mesophyll processes. Such a difference was not observed and even the direction of the average changed for the δ^13^C of older leaves, so it may be a transient effect during development.

Independently of possible wider stomatal apertures of AA compared to FA leaves, the higher transpiration rates, particularly at low D, show higher water consumption of the plants caused by aerosols, including an elevated minimum water loss. These phenomena are similar to the previous investigations for several plant species, which implied that aerosol-induced water loss is not completely under stomatal control. In a study with simultaneous measurement of gas exchange and stomatal pore aperture, NaNO_3_ treatment of *Sambucus nigra* caused higher leaf conductance at the same degree of stomatal opening, with the strongest relative effect at low D (Burkhardt et al., 2001). In a similar study comparing *Vicia faba* grown in ambient and in filtered air, aerosol exposure decreased stomatal apertures at each level of D and increased leaf conductance at comparable levels of aperture (Grantz et al., 2018); at the same time the heterogeneity of pore aperture (“patchiness”) was suppressed, supporting the view that deposition of hygroscopic aerosol may create a thin aqueous film across the leaf surface that connects neighboring stomata to each other and to the leaf interior (Grantz et al., 2020).

A thin aqueous film bridging a stomatal pore along the transverse walls remains the most likely mechanism of enhanced transpiration caused by aerosols. It implies an important impact on hydraulic conductance and can be considered a kind of leakage or bypass not under stomatal control. The hydraulic system ends at the evaporating sites, which are often considered to be the mesophyll cell walls (Rockwell et al., 2014; Wong et al., 2022). When deliquescent material from the leaf surfaces penetrates from outside and connects to the apoplastic liquid water coming from the roots, the new end point of the hydraulic system is translocated to the leaf surface. As compared to water vapor, the liquid water that exits as a thin film along the stomatal pore walls and evaporates on the leaf surface has higher density, is incompressible, and does not respond to changes of stomatal aperture, therefore having the potential to considerably enhance overall transpiration.

**Figure 5:**
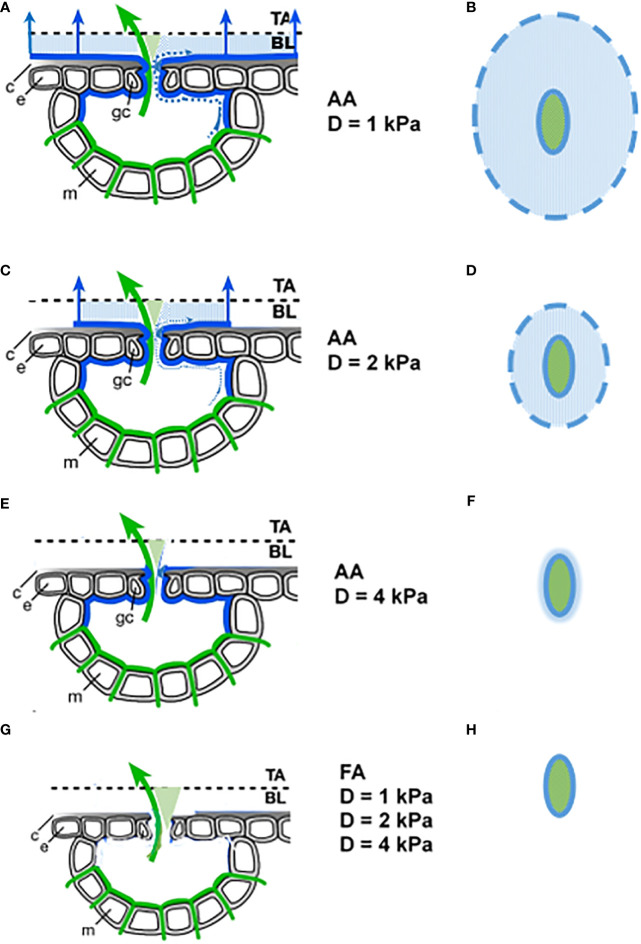
Assumed D dependence of the evaporation process for AA **(A–F)** and FA **(G, H)** leaves. Left column: Section through stomata. Green lines: end of hydraulic system, with evaporation from there shown by green arrow. Blue lines: extension of the hydraulic system by deliquescent deposited aerosols, with additional evaporation from there shown by blue arrows. Dotted blue lines: liquid water that leaves the stomata as a thin film. c cuticle, e epidermal cell, m mesophyll cell, gc guard cell, TA turbulent atmosphere, BL boundary layer. Right column: Top view. Solid lines: stomatal opening. Dashed line: outer end of the wick on the leaf surface. Blue shaded areas are evaporating areas.

Once established, such liquid water connections might persistently act as wicks. Water loss from the outer end of the wick can rapidly be replaced by hydraulic transport from the inner part, and the coupling with atmospheric D becomes more immediate. However, the effectivity of the wick will then depend on the leaf surface area that interacts with the adjacent atmosphere, i.e., where the deliquescence humidity of the hygroscopic surface material is exceeded. The affected leaf area depends on the local leaf surface humidity, which is determined by the humidity of the environment, the distance to stomata and their aperture, as well as the thickness of the leaf boundary layer (Defraeye et al., 2014). Those parts of the wick that are further away from the stomatal pore are the leaf surface areas that will desiccate first in drier conditions and will not further contribute to additional transpiration. Relative humidity at the leaf surface (i.e., the crucial parameter h_s_ in the Ball-Berry equation; Ball et al., 1987) is the most appropriate parameter to describe this process. It is better suited than D, due to the rapid equilibration with hygroscopic salts, which has even been used as a meteorological RH measurement method (Wylie, 1955). This mechanism is also able to explain the stronger HAS effectivity at low D, as illustrated in **Figure 5**.

The upper three rows indicate the AA situation in relatively humid (1 kPa), moderate (2 kPa), and dry (4 kPa) air. A top view of the active surface area is shown in the right column. It becomes smaller with increasing D, until at 4 kPa the additional water loss nearly vanishes and the leaf surface is dry, now approaching the situation that FA leaves experience at any level of D.

The decrease of G with increasing D has often been reported to be exponential or hyperbolic, including a considerable intra- and interspecific variability (Aphalo and Jarvis, 1991; Leuning, 1995; Monteith, 1995; Oren et al., 1999). A frequent assumption is the proportionality between stomatal conductance gs at low D and the sensitivity of the closure response, where sensitivity refers to the magnitude of the reduction in gs with increasing D (Oren et al., 1999). According to this study, G decreased by 65% between D = 1kPa and D = 4kPa (590 mmol m^-2^ s^-1^ to 209 mmol m^-2^ s^-1^) for AA plants and only 20% (270 mmol m^-2^ s^-1^ to 218 mmol m^-2^ s^-1^) for FA plants. Rather than only a decrease of stomatal aperture, this may reflect the reduction of leaf surface area wetted by deliquescence around the stomata, in response to decreasing RH; i.e. a physicochemical rather than a physiological effect.

## Data availability statement

The raw data supporting the conclusions of this article will be made available by the authors, without undue reservation.

## Author contributions

JB: Conceptualization, Funding acquisition, Investigation, Writing – original draft. DZ: Investigation, Methodology, Writing – review & editing. AR: Writing – review & editing. HH: Methodology, Resources, Writing – review & editing. SP: Conceptualization, Investigation, Methodology, Writing – review & editing.
